# Root Canal Morphology of Maxillary Second Molars according to Age and Gender in a Selected Iranian Population: A Cone-Beam Computed Tomography Evaluation

**DOI:** 10.22037/iej.v13i3.19278

**Published:** 2018

**Authors:** Mandana Naseri, Mohammad Ali Mozayeni, Yaser Safi, Maryam Heidarnia, Alireza Akbarzadeh Baghban, Negar Norouzi

**Affiliations:** a * Department of Endodontics, Dental School, Shahid Beheshti University of Medical Sciences, Tehran, Iran; *; b *Department of Oral and Maxillafacial Radioligy, Dental School, Shahid Beheshti University of Medical Sciences, Tehran, Iran; *; c *Dentist, Private Practice, Tehran, Iran; *; d * Physiotherapy Research Center, Rehabilitation School, Shahid Beheshti University of Medical Sciences, Tehran, Iran; *; e *Department of Endodontics, Dental School, Mazandaran University of Medical Sciences, Sari, Iran*

**Keywords:** Age, Cone-Beam Computed Tomography, Gender, Maxillary Second Molar, Root Canal Anatomy, Root Canal Morphology

## Abstract

**Introduction:**

This study sought to assess root canal morphology of maxillary second molars regarding age and gender in an Iranian population using cone-beam computed tomography (CBCT).

**Methods and Materials:**

Totally, 157 maxillary second molars of patients presenting to a radiology clinic were evaluated on CBCT scans. Tooth length, number of roots, root fusion, coronal and sagittal root deviation, number of canals per root, prevalence of second mesiobuccal canal, root canal morphology according to the Vertucci’s classification and the correlation of these variables with age and gender were evaluated. Data were analyzed using the Mann Whitney U, Kruskal Wallis and Fisher’s exact tests.

**Results:**

Of 157 teeth, 98 belonged to females and 59 to males. The mean tooth length was significantly greater in males than in females (*P*=0.002) and it was shorter in 50-60 years old group. The rate of root fusion was 18.6%. Distobuccal and palatal roots were mainly straight in both sagittal and coronal planes while mesiobuccal roots mostly had a distal-buccal deviation; 67.5% of the teeth had four canals. Number of canals was significantly correlated with gender and was higher in males (*P*<0.05). The most prevalent canal type was type VI in second mesiobuccal, and type V in palatal and distobuccal canals. The most common types in mesiobuccal canal were types I, VI and II, respectively. In the remaining two roots, type I was the most common.

**Conclusion:**

Root and canal morphology of the maxillary second molars in Iranian population showed features different from those in other populations.

## Introduction

A successful endodontic treatment requires a thorough knowledge of tooth anatomy and morphology of the root canal system because there is a wide variability in this respect even within the normal range [1]. Inadequate knowledge in this regard will lead to incomplete debridement and filling of the root canals, which is the main cause of failure of root canal treatments [2]. The external morphology and internal anatomy of the teeth are highly variable in terms of number and shape of roots and canals [1]. Morphological variations in root canal anatomy due to ethnicity and genetic differences have been reported in many studies [3, 4]; therefore, it is required to identify root canal anatomy of different populations for successful endodontic treatment [5]. 

Several methods have been suggested for evaluation of root canal morphology. Cone-beam computed tomography (CBCT), introduced to endodontics in 1990, is suggested for assessment of anatomy and morphology of the root canal system [6], since it provides 3D images of tooth structure with no destruction and enables thorough assessment of the internal and external morphology of the root canal system [7, 8]. Compared to micro-CT with limited application for extracted teeth or pieces of the jaw with teeth [9], CBCT is applicable for use in patients and for all teeth. Comparing the evaluation of tooth anatomy by CBCT and conventional periapical radiography revealed that measurement of tooth length on CBCT scans was at least as reliable and accurate as that on periapical [10] and more accurate than panoramic radiography [11]. Due to the above-mentioned advantages, several studies have recommended CBCT as an accurate and reliable modality for evaluation of root canal anatomy [12-14]. 

**Figure 1:**
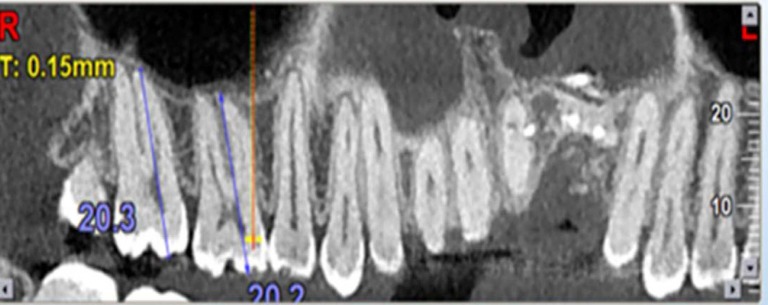
Measurement of tooth length

**Figure 2:**
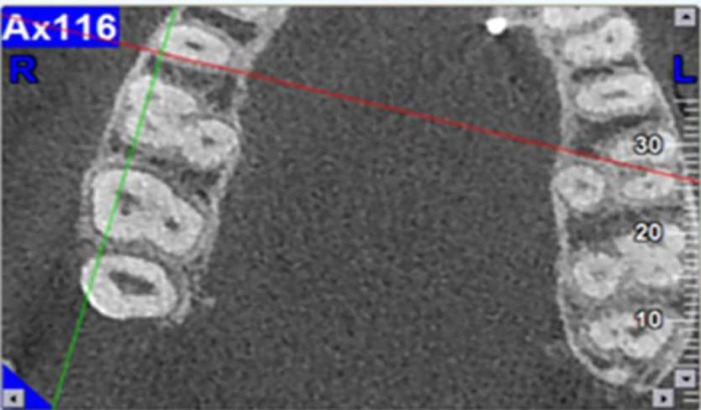
Assessment of root fusion

Reviews on the applications of CBCT in endodontics show that CBCT with a small field of view, high resolution and low patient radiation dose can be used to assess root canal morphology [15], with high reliability for image reconstruction of the root canal system, compared with CBCT scans with histological sections [16]. Compatibility of CBCT with histological sections is higher than periapical radiographs [17, 18], suggesting it as an efficient and reliable technique to overcome the limitations of conventional radiography [19]. This technique enables collecting data based on age, gender and position of the tooth [20] and is an acceptable modality for assessment of the presence of second mesiobuccal canal compared to the gold standard;* i.e. *tooth sectioning [7]. 

The aim of this study was to evaluate the root and canal morphology of maxillary second molars regarding age and gender in an Iranian population using CBCT. 

## Materials and Methods

This retrospective, descriptive study was conducted in Dental Imaging Center of Shahid Beheshti Dental School in 2014. A total of 157 maxillary second molars were evaluated on CBCT scans of patients obtained for surgical procedures, implant therapy or orthodontic treatment. 

The inclusion criteria were optimal quality of CBCT scans, showing the maxillary second molar area with no artifacts and age of over 15 years old, for the apex of this tooth being fully formed. The exclusion criteria were the patients younger than 15 years old, congenital missing or extraction of this tooth, root resorption, calcification and endodontically treated teeth. A total of 250 CBCT scans were primarily evaluated. After applying the inclusion and exclusion criteria, 157 maxillary second molars remained in the study. 

All CBCT scans were taken using NewTom VGi CBCT unit (QR SRL Company, Verona, Italy) and analyzed with NewTom NNT viewer version 5.3 software (Quantitative Radiology, Verona, Italy). CBCT scan’s parameters were 8×12 cm field of view (FOV), 200 µm voxel size, 14 mA, 90 kVp, exposure time of 3.6 sec, and 15-bit grayscale for the purpose of standardization. Patients were evaluated in two groups of males and females and in six age groups of 15-20, 20-30, 30-40, 40-50, 50-60 and 60-70 years old. 

***Measurement of tooth length***

The object cursor was adjusted to the longitudinal axis of the tooth to obtain the highest clarity. Once this axis was parallel to the sagittal plane, tooth length was measured from the apex of the longest root to the tip of the mesiobuccal cusp using the software ruler with 0.1 mm accuracy (Figure 1).

***Number of roots***

To assess the number of roots, mesiobuccal, distobuccal and palatal roots were evaluated on the sagittal sections. Fusion, if present, could be seen on the sagittal sections and also the reconstructed panoramic image by the CBCT unit. In addition, evaluation of the axial section of the roots enabled the detection of fourth root or fused roots, if present (Figure 2).

***Canal type***

The type of canals in each root was assessed on coronal and axial sections. By observing the axial sections, number of orifices, canal path and number of apical foramina were assessed. Canal path was also evaluated on coronal planes. The type of each canal was determined according to the Vertucci’s classification [21].

***Deviation of the roots/the apical foramina from root apex***

Each tooth was evaluated on sagittal and coronal planes. Evaluation of the teeth on coronal and sagittal sections revealed buccal/labial and mesial/distal deviation of the roots and apical foramina, respectively. 

***Statistical analysis***

Descriptive statistics of age and gender were calculated. The CBCT scans were selected using convenience sampling. Sample size was calculated to be 157 assuming 95% confidence interval, *δ*=6.0 [2] and *d*=0.05.

The data were analyzed using the independent *t*-test, one sample Kolmogorov-Smirnov test, Levene’s test, one-way ANOVA, Mann Whitney test, Kruskal Wallis test, Fisher’s exact test, McNemar’s test, Wilcoxon signed rank test and marginal homogeneity test. Statistical analysis was performed using the SPSS software (SPSS version 21.0, SPSS, Chicago, IL, USA). The level of significance was set at 0.05.

***Ethical considerations***

This study was done using CBCT archive images, therefore no ethical considerations were taken into account.

## Results

A total of 157 maxillary second molars were evaluated, out of which 98 (62.4%) belonged to female and 59 (37.6%) to male patients. The maxillary second molars of both sides were evaluated in 6 patients. Patients were in the age range of 15 to 70 years old with the highest frequency of 20-30 years old (*n*=42, 26.8%).

***Tooth length***

The mean tooth length was 19.6±0.16 mm in female and 20.5±0.24 mm in male patients. Independent *t*-test showed that the mean tooth length was significantly greater in males than in females (*P*=0.002). This measure was 19.92 mm and 20.14 mm in right and left teeth, respectively. The difference in this regard was not significant (*P*=0.135). 

One-sample Kolmogorov-Smirnov test showed that tooth length data were normally distributed in the six age groups (*P*>0.05). Equality of variances was also confirmed by the Levine’s test (*P*=0.180). One-way ANOVA revealed significant differences in tooth length among the six age groups (*P*<0.05). Pairwise comparisons by Tukey’s test revealed that the shortest tooth length belonged to the 50-60 years old group (*P*<0.001). 

***Fusion***

According to the Fisher’s exact test, the difference between males and females (*P*=0.055) or different age groups (*P*=0.613) in terms of root fusion was not statistically significant. The frequency of root fusion (18.6%) was not significantly different between the right and left sides (McNamar’s test, *P*=0.687). 

**Table 1:** Root deviation of maxillary second molar in coronal and sagittal views

		**Straight/ straight**	**Straight/ distal**	**Straight/ mesial**	**Buccal/ straight**	**Buccal/ distal**	**Buccal/mesial**	**Palatal/ straight**	**Palatal/distal**	**Palatal/mesial**
**Root **	**Mesiobuccal**	9	10	0	6	40	1	0	1	1
	**Distobuccal**	42	4	4	6	5	0	3	1	3
	**Palatal**	32	9	1	9	10	0	3	0	2

**Table 2:** Distribution of number of canals in maxillary second molar according to gender and age

**Gender and age group (years)**	**Number of canals (%)**			**Total**
	**3 **	**4**	**5**	
**Males**	12 (20.3%)	46 (78%)	1 (1.7%)	59 (100%)
**Females**	37 (37.8%)	60 (61.2%)	1 (1.0%)	98 (100.0%)
**15-20 **	5 (55.6%)	4 (44.4%)	0 (0%)	9 (100.0%)
**20-30 **	12 (28.6%)	29 (69.0%)	1 (2.4%)	42 (100.0%)
**30-40 **	15 (37.5%)	25 (62.5%)	0 (0%)	40 (100.0%)
**40-50 **	5 (20.0%)	20 (80.0%)	0 (0%)	25 (100.0%)
**50-60 **	6 (27.3%)	15 (68.2%)	1 (4.5%)	22 (100.0%)
**60-70 **	6 (31.6%)	13 (68.4%)	0 (0%)	19 (100.0%)
**Total**	49 (31.2%)	106 (67.5%)	2 (1.3%)	157 (100.0%)

***Prevalence of root deviation***

Mesiobuccal roots mostly had a distal-buccal deviation. According to the Fisher’s exact test, no significant difference existed in the coronal and sagittal planes between males and females (*P*=0.359 and 0.710) or different age groups (*P*=0.154 and 0.068) in terms of mesiobuccal root deviation. 

Distobuccal and palatal roots were mainly straight in both sagittal and coronal planes. No significant difference existed in distobuccal root deviation in the coronal plane between males and females (*P*=0.137) or different age groups (*P*=0.162). The difference in the deviation of palatal root in the coronal plane was not significant either between males and females (*P*=0.161) or different age groups (*P*=0.532). This difference in the sagittal plane was not significant either (*P*=0.801 and* P*=0.185 for the comparison between males and females and different age groups, respectively). The frequency distribution of mesiobuccal, distobuccal and palatal root deviations in the coronal and sagittal planes is presented in Table 1.

***Number of canals***

The frequency and percentage of root canal numbers according to gender and age is presented in Table 2. The Mann Whitney test showed a significant difference in the number of root canals between male and female patients (*P*=0.023) and was greater in males. The Kruskal Wallis and Wilcoxon tests found significant relation neither between the number of canals and age nor between the position of the tooth and age (*P*>0.05 and *P*=0.819, respectively). 

***Vertucci classification of canal pattern***

Fisher’s exact test showed no significant difference between males and females in terms of mesiobuccal canal type (*P*=0.054). 

However, the difference in this regard among different age groups was significant (*P*=0.011). In the age group of 15-20 years, 56% of root canals were type I while in the age group of 20-30 years, 14% and 36% were type V and VI, respectively. 

The difference in distobuccal canal type between males and females (*P*=0.264) or different age groups (*P*=0.547) was not significant. The same was observed for palatal canal (*P*=0.589 and 0.550).

Marginal homogeneity test showed that the right and left quadrants were not significantly different in terms of distribution of mesiobuccal (*P*=0.470), distobuccal (*P*=0.408) and palatal canal types (*P*=0.490). Tables 3 to 5 show the frequency distribution of the types of mesiobuccal, distobuccal and palatal root canals based on age and gender. 

**Table 3:** Frequency distribution of mesiobuccal root canal type in males and females and different age groups

		**Number (%)of Canal type**						
		**I**	**II**	**III**	**IV**	**V**	**VI**	**Total**
**Gender**	**Male**	12 (20.3)	16 (27.1)	2 (3.4)	6 (10.2)	3 (5.1)	20 (33.9)	59 (100)
	**Female**	39 (39.8)	13 (13.3)	3 (3.1)	12 (12.2)	9 (9.2)	22 (22.4)	98 (100)
**Age (year)**	**15-20 **	5 (55.6)	1 (11.1)	0 (0)	3 (33.3)	0 (0)	0 (0)	9 (100)
	**20-30 **	12 (28.6)	5 (11.9)	3 (7.1)	1 (2.4)	6 (14.3)	15 (35.7)	42 (100)
	**30-40 **	15 (37.5)	8 (20.0)	2 (5)	3 (7.5)	3 (7.5)	9 (22.5)	40 (100)
	**40-50 **	6 (24.0)	11 (44)	0 (0)	2 (8)	1 (4.0)	5 (20.0)	25 (100)
	**50-60 **	7 (31.8)	2 (9.1)	0 (0)	3 (13.6)	1 (4.5)	9 (40.9)	22 (100)
	**60-70**	6 (31.6)	2 (10.5)	0 (0)	6 (31.6)	1 (5.3)	4 (21.1)	19 (100)
**Total**		51 (32.5)	29 (18.5)	5 (3.2)	18 (11.5)	12 (7.6)	42 (26.8)	157 (100)

**Table 4:** Frequency distribution of distobuccal root canal types in males and females and different age groups

**Canal type**		**I**	**III**	**V**	**VI**	**Total**
**Gender**	**Females**	55 (93.2)	0 (0)	4 (6.8)	0 (0)	59 (100)
	**Males**	93 (94.9)	1 (1)	2 (2)	2 (2)	98 (100)
**Age (year)**	**15-20 **	9 (100)	0 (0)	0 (0)	0 (0)	9 (100)
	**20-30 **	40 (95.2)	0 (0)	1 (2.4)	1 (2.4)	42 (100)
	**30-40**	39 (97.5)	0 (0)	1 (2.5)	0 (0)	40 (100)
	**40-50 **	22 (88)	1 (4)	2 (8)	0 (0)	25 (100)
	**50-60 **	20 (90.9)	0 (0)	2 (9.1)	0 (0)	22 (100)
	**60-70 **	18 (94.7)	0 (0)	0 (0)	1 (5.3)	19 (100)
**Total**		148 (94.3)	1 (6)	6 (3.8)	2 (1.3)	157 (100)

***Apical foramen deviation from the anatomic apex***

The apical foramen of mesiobuccal root was mainly straight (50%) in the coronal and with a distal deviation (56.7%) in sagittal plane. The Fisher’s exact test found no significant difference between males and females (*P*=0.151) or different age groups (*P*=0.557) in terms of apical foramen deviation of the mesiobuccal canal in the coronal plane. There was no difference in this regard for the mesiobuccal canal apical foramen in the sagittal plane (*P*=0.626 and *P*=0.615 for the comparison of males and females and age groups, respectively).

The apical foramen of distobuccal and palatal roots were mainly straight in both sagittal and coronal planes. There was no significant difference between males and females (*P*=0.689) or age groups (*P*=0.492) for apical foramen deviation of distobuccal canal in the coronal plane. Using Fisher’s exact test, no significant difference was noted between males and females (*P*=0.332) or different age groups (*P*=0.525) in apical foramen deviation of distobuccal canal in the sagittal plane. Significant difference was noted neither between males and females (*P*=0.787) nor different age groups (*P*=0.144) in apical foramen deviation of palatal canal in the coronal plane. However, the difference between males and females in frequency distribution of apical foramen deviation of palatal canal in the sagittal plane was statistically significant (*P*=0.043), as in 41% of females, apical foramen of palatal canal was straight in the sagittal plane, while this rate was 59% in males. The difference in apical foramen deviation of the palatal canal in the sagittal plane among different age groups was not significant (*P*=0.369).

## Discussion

Finding and accessing the root canals is fundamental for a successful endodontic treatment. Inadequate knowledge about the anatomy of the root canals is a major cause of treatment failure [22]. The results of previous studies on the anatomy of the teeth and pulp are controversial. Studies on the internal and external anatomy of teeth have shown that complex anatomical variations may occur in all teeth [23, 24]. Many factors play a role in these variations in root canal anatomy such as ethnicity [25, 26], age [27], gender [28] and study design (*in vitro* *versus* *in vivo*) [26]. Since the maxillary molars have often a complex anatomy, in this study, the anatomy of maxillary second molars was evaluated in an Iranian population. This is one of the few and the first Iranian study that evaluated the relation of anatomy and gender or age of patients.

All maxillary second molars evaluated in this study had three roots, similar to previous studies on Iranian populations: Naseri *et al.* [20] also reported three roots in 100% of patients, which is identical to our results, while Rohani *et al*. [5] and Khademi *et al.* [29] reported three roots in 98.4% and 93.5% of patients, respectively. Studies on Taiwanese, Kuwaiti, Chinese and Burmese populations also showed that all maxillary molars had three roots [30-33]. On the other hand, studies on Brazilian, Indian and Korean populations reported that 4-25% of maxillary molars did not have three roots [2, 7, 14, 34]. These differences in root canal anatomy may indicate the effect of ethnicity on root canal morphology [35]. In the current study, fusion of the roots was seen in 18.6% of the cases, confirming those of previous studies [14, 31, 36]. Other studies have reported a fusion rate of about 8% [2, 5]. As to a review on 6 studies reporting root fusion in this tooth, ethnicity plays a role in different rates reported and the lowest frequency of root fusion in this tooth is reported in Iranian and the highest in Brazilian population [35]. Comparison of root fusion with gender, in the present study, showed no significant difference between males and females or different age groups, in this respect, while a Chinese study showed different frequency and form of root fusion between males and females, explained by different cementum deposition with time [37]. These differences can also be explained by the ethnologic differences of tooth morphology in different populations. 

**Table 5:** Frequency distribution of palatal root canal types in males and females and different age groups

		**Number (%) of Canal type**				
		**I**	**III**	**V**	**VI**	**Total**
**Gender**	**Females**	54 (91.5)	1 (1.7)	3 (5.1)	1 (1.7)	59 (100)
	**Males**	93 (94.9)	0 (0)	4 (4.1)	1 (1)	98 (100)
**Age (year)**	**15-20 **	9 (100)	0 (0)	0 (0)	0 (0)	9 (100)
	**20-30 **	36 (85.7)	1 (2.4)	4 (9.5)	1 (2.4)	42 (100)
	**30-40 **	40 (100)	0 (0)	0 (0)	0 (0)	40 (100)
	**40-50 **	23 (92.0)	0 (0)	2 (8.0)	0 (0)	25 (100)
	**50-60 **	22 (100)	0 (0)	0 (0)	0 (0)	22 (100)
	**60-70 **	17 (89.5)	0 (0)	1 (5.3)	1 (5.3)	19 (100)
**Total**		147 (93.6)	1 (6)	7 (4.5)	2 (1.3)	157 (100)

The results of this study regarding the length of maxillary second molars showed that the mean length of this tooth was significantly greater in males compared to females (20.5 mm versus 19.6 mm). Similar results were reported by Naseri and colleagues (2016) (mean tooth length of 19.3 in females and 20.3 mm in males) [20]. A similar mean was reported in an Indian [38] and Brazilian study [2], suggesting that the mean length of maxillary second molars are about the same in different populations. Also, in the present study, tooth length was shorter in 50-60 years old than that in other age groups, while in the study by Naseri *et al.* [20], teeth length was not associated with age. Due to the alterations of tooth morphology by age, we think that the results of our study is more valid. In this study, deviation of the root and apical foramen was evaluated in two dimensions of sagittal and coronal. The majority of mesiobuccal roots had distal-buccal deviation. Mesiobuccal root deviation in the sagittal plane was mainly distally, and straight roots had a lower prevalence. It was mainly straight in the coronal plane as well. Distobuccal and palatal roots were mainly straight in both sagittal and coronal planes. The apical foramen of mesiobuccal root in the coronal plane was mainly straight (50%). In the sagittal plane, it mainly had a distal deviation (56.7%). Distobuccal root apical foramen was mainly straight in the coronal plane (51%). In the sagittal plane, it was mainly straight (47.8%). In the palatal root, the apical foramen was mainly straight in the coronal plane (45.2%). In the sagittal plane, it was mainly straight (48.4%). Straight apical foramen of the palatal root in the sagittal plane had a higher prevalence in males. No other significant associations were noted between root and apical foramen deviation and gender. Naseri *et al.* [20], also reported that all three roots were straight in coronal plane, and in sagittal plane, mesiobuccal root deviations were mainly distal and distobuccal and palatal roots were straight [20], which matches our results. Nonetheless, Vertucci [21] showed that in mesiobuccal, distobuccal and palatal roots, apical foramen was straight in 12%, 17% and 19% of the cases, respectively, which is different than our results. This difference can be justified by different methods of evaluation, since staining was the method used by Vertucci. 

The results of the current study showed that according to the Vertucci’s classification [21], mesiobuccal root was single-canal (type I) in 32.5% and had two canals in 67.5%. In cases with two canals, type VI (26.8%), followed by type II (18.5%), were the most common. In the remaining two roots, type I had the highest prevalence (94.3% of distobuccal and 93.6% of palatal roots). In previous studies, type I canal had higher prevalence in mesiobuccal root while palatal and distobuccal roots with more than one canals were more prevalent [2, 34, 36]. Silva *et al.* [2] showed that 45.09% of second molars had three roots and one canal per each root; 34.32% had three roots with one canal in each of the palatal and distobuccal roots and two canals per each mesiobuccal root. In the study by Rohani *et al.* [5], type I morphology had the highest prevalence (80.8%) in distobuccal and palatal roots, which was similar to the results of the current study. Pawar *et al.* [38] also reported type I as the predominant canal configuration in distal and extra roots and type IV as the most common in mesial roots, which is contrary to the results of the present study, although it confirms the high possibility of presence of two canals in the mesiobuccal root of the maxillary second molars, which is an important finding that has to be taken into account for a successful endodontic treatment in the clinical setting. Comparing the mesiobuccal roots with two canals between the first and the second molar has shown a more complex system in the second molar [39], which adds to the significance of paying attention to this issue in this tooth. 

In this study, no association was found between canal type and gender, which was in line with the findings of previous studies [14, 21, 36]. But in the age group of 15-20 years, the most common canal type was type VI. Fernandes *et al.* [40] reported no association between two canals in mesiobuccal root and patients age or gender. In the current study the number of root canals was significantly greater in males than that in females, but it had no significant relation with patients’ age. Kim *et al.* [34] reported that the prevalence of second mesiobuccal canal was higher in males, while it had no significant correlation with age or tooth position, which is consistent with the results of the present study indicating higher root canals in male patients. Additionally, the current study showed that the root canal system (type and number of canals per each root) was not significantly different in the right and left quadrants, which was in agreement with the results of Kim *et al*. [34], indicating that the root canal system of maxillary second molars was the same in both sides in 82% of the cases. 

Evaluation of a relatively large sample size was the main strength of this study. But, due to ethical considerations, we were only allowed to use the CBCT scans already taken for other purposes, which served as the main limitation of the present study. 

## Conclusion

Root canal morphology of the maxillary second molars was widely variable in our sample of Iranian population, and the prevalence of anatomical variations was different from that in other populations. 
